# Efficient hydrogen production from the lignocellulosic energy crop *Miscanthus *by the extreme thermophilic bacteria *Caldicellulosiruptor saccharolyticus *and *Thermotoga neapolitana*

**DOI:** 10.1186/1754-6834-2-12

**Published:** 2009-06-17

**Authors:** Truus de Vrije, Robert R Bakker, Miriam AW Budde, Man H Lai, Astrid E Mars, Pieternel AM Claassen

**Affiliations:** 1 Agrotechnology and Food Sciences Group, Wageningen University and Research Centre, PO Box 17, 6700 AA Wageningen, the Netherlands

## Abstract

**Background:**

The production of hydrogen from biomass by fermentation is one of the routes that can contribute to a future sustainable hydrogen economy. Lignocellulosic biomass is an attractive feedstock because of its abundance, low production costs and high polysaccharide content.

**Results:**

Batch cultures of *Caldicellulosiruptor saccharolyticus *and *Thermotoga neapolitana *produced hydrogen, carbon dioxide and acetic acid as the main products from soluble saccharides in *Miscanthus *hydrolysate. The presence of fermentation inhibitors, such as furfural and 5-hydroxylmethyl furfural, in this lignocellulosic hydrolysate was avoided by the mild alkaline-pretreatment conditions at a low temperature of 75°C. Both microorganisms simultaneously and completely utilized all pentoses, hexoses and oligomeric saccharides up to a total concentration of 17 g l^-1 ^in pH-controlled batch cultures. *T. neapolitana *showed a preference for glucose over xylose, which are the main sugars in the hydrolysate. Hydrogen yields of 2.9 to 3.4 mol H_2 _per mol of hexose, corresponding to 74 to 85% of the theoretical yield, were obtained in these batch fermentations. The yields were higher with cultures of *C*. *saccharolyticus *compared to *T. neapolitana*. In contrast, the rate of substrate consumption and hydrogen production was higher with *T. neapolitana*. At substrate concentrations exceeding 30 g l^-1^, sugar consumption was incomplete, and lower hydrogen yields of 2.0 to 2.4 mol per mol of consumed hexose were obtained.

**Conclusion:**

Efficient hydrogen production in combination with simultaneous and complete utilization of all saccharides has been obtained during the growth of thermophilic bacteria on hydrolysate of the lignocellulosic feedstock *Miscanthus*. The use of thermophilic bacteria will therefore significantly contribute to the energy efficiency of a bioprocess for hydrogen production from biomass.

## Background

In view of the transition to hydrogen as a major energy carrier in the future, new routes for hydrogen production need to be explored. The production of hydrogen from biomass is one of the options for contributing to the supply of exploitable renewable resources. Hydrogen can be produced from a vast range of biomass, using thermochemical, as well as fermentative, processes. Carbohydrates, such as sugars, starch or (hemi)cellulose, are the prime substrates for fermentative processes. For future sustainability of the energy supply, the utilization of (hemi)cellulose is of prime interest, as this component is most abundant in crops that can be grown for the purpose of energy supply.

To date, many studies have been done on fermentative hydrogen production from pure sugars and from feedstocks, such as by-products from the agricultural and food industry, municipal waste, or wastewaters [1]. However, only a few studies describe the production of hydrogen from lignocellulosic biomass (reviewed in [2]). Some of these feedstocks were offered as solid materials, such as office paper waste [3], wheat straw waste [4], delignified wood fibres [5], and a variety of cellulosic waste materials [6]. Other types of biomass, such as *Miscanthus *[7], paper sludge [8], corn stover [9], and corn stalks [10] were first pretreated and/or hydrolyzed to obtain a soluble substrate of mixed sugars and oligosaccharides. Bacterial consortia from anaerobic digester sludge or cow dung compost, as well as single cultures of mesophilic (*Clostridium acetobutylicum*) and thermophilic bacteria (*Clostridium thermocellum*, *Caldicellulosiruptor saccharolyticus *and *Thermotoga elfii*), have been used as hydrogen producers. The reported hydrogen yields on these lignocellulosic substrates varied greatly from approximately 10% to more than 80% of the theoretical value, which is 4 mol of hydrogen per mol of hexose. The diversity of the applied feedstocks and pretreatment methods hardly allow a comparison of hydrogen production efficiency.

It is proposed that research should focus on the conversion of biomass to fermentable substrates to obtain a successful introduction of cellulosic biomass for the production of biofuels [11]. The fermentability of lignocellulose is improved by pretreatment of the biomass. This is required to overcome the recalcitrance of the lignocellulosic complex by altering its structure, which makes the cellulose and hemicelluloses accessible to the enzymes [12]. One of the methods of interest is pretreatment with an alkaline agent at relatively low temperatures (<100°C). One of the main effects of the alkali is the disruption of the intermolecular bonding between xylan and other biomass components, such as lignin or other hemicellulosic components, resulting in increased porosity of the lignocellulosic biomass. In addition, applying alkali in a water solution generally leads to swelling of cell wall material, thereby increasing the internal surface of the lignocellulosic matrix. These effects (that is, increase of porosity; swelling of fibrous material) lead to an increase in enzymatic degradability, as the lignocellulosic matrix is more accessible for enzymes. Another effect, less well understood, is the modification of lignin during alkaline pretreatment. Depending on the alkali used and the biomass type, alkaline pretreatment may lead to lignin depolymerisation, and (partial) dissolution of lignin components.

This study describes the efficiency of hydrogen production from *Miscanthus*, a lignocellulosic energy crop. *Miscanthus *is a rapidly growing perennial C_4 _grass with relatively high yields of 8 to 15 ton dry weight per ha in Western European regions, which requires only low inputs of nutrients for cultivation. It has been studied for years because of its potential for future energy supply. Previously, we have compared mechanical methods of pretreatment, milling and extrusion, in combination with chemical treatment. The applied methods were aimed at the high conversion of polysaccharides and high yields of monomeric sugars [7]. In the present study, the effect of various chemicals used for alkali pretreatment on the fermentability of the feedstock is investigated. For this, two extreme thermophilic bacteria were used, the cellulolytic bacterium *Caldicellulosiruptor saccharolyticus *of the order Clostridiales [13], and the moderately halophilic bacterium *Thermotoga neapolitana *of the order Thermotogales [14], which grow at optimum temperatures of 70 and 80°C, respectively. Thermophilic bacteria are superior with respect to hydrogen yield [15-17], due to favourable thermodynamical conditions at high temperatures, and reduced variety in by-product formation. Furthermore, many thermophilic bacteria, including *T. neapolitana *and *C. saccharolyticus*, are able to utilize a wide range of substrates for growth from simple sugars to complex carbohydrates [18,19]. Unlike *T. neapolitana*, *C. saccharolyticus *is capable of growth on crystalline cellulose [13], and on lignocellulosic feedstocks, although degradation of these substrates was limited [20]. Therefore, the pretreatment was followed by enzymatic hydrolysis of *Miscanthus *to prepare fermentable substrates. The soluble fraction, that is, the hydrolysate, will be a complex mixture of monomeric C6 and C5 sugars, and di- and oligosaccharides. The production of hydrogen from all of these sugars is required to develop an efficient process. The results showed that both thermophiles were able to consume most, if not all, soluble carbohydrates present in the *Miscanthus *hydrolysate. Hydrogen was produced at high yields of more than 75% of the theoretical value.

## Results and discussion

### Comparison of alkaline pretreatment methods

Two types of alkali, NaOH and Ca(OH)_2_, were tested for the pretreatment of *Miscanthus*. The dosage of these types of alkali was based on earlier reported work [7,21]. Indicators of the efficiency of lignocellulosic biomass pretreatment are the pH after the addition of the alkali at the start of the pretreatment at 85°C, the amount of delignification after pretreatment, and the enzymatic degradability of the pretreated biomass. The results showed that NaOH was most effective because of the higher initial pH of the biomass, and the lower lignin content of the insoluble fraction of the material that was pretreated with NaOH (Table 1). Still, a reasonable amount of monomeric sugars can be released from Ca(OH)_2_-pretreated *Miscanthus*, despite the insignificant delignification of the biomass during pretreatment (Table 2).

**Table 1:** Effect of alkaline pretreatment on *Miscanthus *biomass.

Alkali	pH	Insoluble fraction	Lignin, % of insoluble fraction	
		% of initial dry matter	Acid-insoluble	Acid-soluble
None	-	100	21.9	0.8
Ca(OH)_2_	10.8	87	24.4	0.8
NaOH	11.5	64	12.9	0.6

The pH of *Miscanthus *biomass after the addition of 0.10 g g^-1 ^Ca(OH)_2 _or 0.09 g g^-1 ^NaOH at the start of pretreatment at 85°C for 16 h, and the fraction recovered as insoluble material after pretreatment with the lignin content.

**Table 2:** Effect of different methods for alkaline pretreatment and enzymatic hydrolysis on the *Miscanthus *hydrolysate composition.

		Hydrolysate			
		I	II	III	IV^a^
Pretreatment	Alkali	Ca(OH)_2_	Ca(OH)_2_	NaOH	NaOH
	g g^-1^	0.10	0.10	0.12	0.09
	mM	157	164	334	265
	pH_initial_	10.4	10.2	11.1	11.0

pH adjustment	Acid	H_2_SO_4_	H_3_PO_4_	CH_3_COOH	CH_3_COOH
	mM	123	88	302	123
	pH_end_	4.8	5.1	4.9	4.8

Enzymatic hydrolysis	Enzyme 1	Cellubrix	GC220	Cellubrix	GC220
	Enzyme 2	Novozym 188	-	Novozym 188	-

Hydrolysate composition					
Sugars (g l^-1^)	Arabinose	1.8	1.5	1.6	1.1
	Galactose	0.3	0.2	0.2	0.0
	Glucose	26.3	19.9	33.0	29.0
	Xylose	11.5	7.6	11.1	10.0
*Total carbohydrates*		*39.9*	*29.1*	*46.0*	*40.1*
Organic acids	Acetic acid^b^	84	62	419	130
(mM)	Lactic acid^c^	12	10	14	1.1

^a^During preparation of hydrolysate IV the alkali pretreated material was washed 3 times with water (5.5 l l^-1^) prior to pH adjustment. ^b^Acetic acid was used for pH adjustment and is also a product of biomass deacylation. ^c^Lactic acid is formed by contaminating fermentative microorganisms during enzymatic hydrolysis.

Pretreatment of 205–210 g of dry matter of *Miscanthus *(2 mm sieve) was done at a solid content of 0.125 g g^-1 ^for 16 h at 85°C. Enzymatic hydrolysis was carried out at 50°C for 24 h.

The selection of an optimal pretreatment method will not only be determined by the efficiencies of the biomass pretreatment, and the enzymatic hydrolysis of the polysaccharides, but also by the fermentability of the hydrolysates. Hydrolysates were prepared from batches of approximately 200 g of milled *Miscanthus*, which were pretreated using NaOH or Ca(OH)_2_, titrated with different acids prior to enzymatic hydrolysis, and hydrolyzed with commercial enzyme preparations, after which the sugar composition of the hydrolysates was determined (Table 2). In addition, the acid usage in the preparation of hydrolysate IV was reduced by circa 50% through washing with water prior to pH adjustment. Glucose and xylose were the main monosaccharides in the hydrolysates. The highest amount of monomeric sugars was found in hydrolysate III, which has been prepared by pretreatment with the highest amount of alkali. This resulted in a polysaccharide conversion efficiency of circa 55%.

### Fermentability of hydrolysates on small scale and inhibitory compounds

The fermentability of the four hydrolysates was tested in small-scale experiments using closed flasks. For this, the production of hydrogen and the main organic acids (acetate and lactate) by *C. saccharolyticus *and *T. neapolitana *on a concentration range of diluted hydrolysate was monitored and compared to production in control fermentations using pure sugars. The final sugar concentration in all samples was adjusted to 10 g l^-1 ^by adding pure sugars (glucose and xylose, 7:3, w w^-1^). Both bacteria were able to grow and produce hydrogen with all hydrolysates (Table 3). *C. saccharolyticus *performed well on hydrolysates II and IV. The fermentability of hydrolysate I, and to a lesser extent hydrolysate III, was poor as production of organic acids was already inhibited at low sugar concentrations. *T. neapolitana *also showed good growth and organic acid production during growth on hydrolysate IV, and fermentation was slightly inhibited with hydrolysate I. In contrast to *C. saccharolyticus*, organic acid production was inhibited on hydrolysate II.

**Table 3:** Fermentability of *Miscanthus *hydrolysates by *C. saccharolyticus *and *T. neapolitana*.

	Hydrolysate			
Microorganism	I	II	III	IV
	IC_20 _(g sugars l^-1^)^a^			
*C. saccharolyticus*	5 to 7.5	+	7.5 to 10	+
*T. neapolitana*	7.5 to 10	7.5	n.d.	+

^a^IC_20_, hydrolysate concentration (g monomeric sugars l^-1^) at which 20% inhibition of organic acid production (acetic and lactic acid) occurred compared to a control fermentation on pure sugars; +, stimulation of organic acid production at the highest hydrolysate concentration tested (10 g monomeric sugars l^-1^); n.d., not determined.

Diluted hydrolysates were tested in a concentration range. The final sugar concentration in all samples was adjusted to 10 g l^-1 ^by addition of pure sugars. The production of organic acids (acetate and lactate) was monitored and compared to production in the control fermentations using pure sugars.

The inhibition of fermentation might be caused by the chemicals used for pretreatment and pH adjustment. Growth of *C. saccharolyticus *is inhibited by high ionic strength of the culture medium [22]. Indeed, 50% inhibition of growth and the production of hydrogen and organic acids were observed at approximately 130 mM acetate (Table 4). This effect was not observed with *T. neapolitana*, which is in line with its moderately halophilic nature. The high concentration of acetate in hydrolysate III probably caused the reduced organic acid production with *C. saccharolyticus*. The washing of the alkaline-pretreated material prior to pH adjustment reduced the use of acetic acid (Table 2) and prevented the inhibition of fermentation (Table 3).

**Table 4:** Effect of chemicals on the growth of *C. saccharolyticus *and *T. neapolitana *and the production of hydrogen and organic acids (acetic and lactic acid).

Component		*C. saccharolyticus*	*T. neapolitana*
		IC_50_	
Sodium acetate	mmol l^-1^	130	>250
Calcium chloride	mmol l^-1^	10 to 30	>50^a^
Potassium phosphate	mmol l^-1^	35	35 to 50
Sodium sulfate	mmol l^-1^	35	>50
Furfural	g l^-1^	1 to 2	2 to 4
HMF	g l^-1^	1 to 2	2 to 4

^a^The presence of CaCl_2 _in the culture medium stimulated the production of hydrogen and organic acids up to a concentration of 50 mmol l^-1^.

IC_50 _inhibitor concentration (mmol l^-1 ^or g l^-1^), at which 50% inhibition of growth or production occurred.

Salts, such as calcium chloride, potassium phosphate, and sodium sulfate, also inhibit *C. saccharolyticus *(Table 4). The presence of calcium and sulfate ions in hydrolysate I (Table 2) probably contributed to the strong inhibition with this hydrolysate. The inhibition of *C. saccharolyticus *was not observed with hydrolysate II, because the concentration of calcium ions was reduced through formation of an insoluble salt when phosphoric acid, instead of sulfuric acid, was used for pH adjustment. The growth of *T. neapolitana *appeared to be stimulated by the presence of calcium ions up to 50 mmol l^-1^, and the organism is less sensitive for phosphate and sulfate (Table 4). The inhibition of fermentation by hydrolysate I and II is, therefore, likely to be caused by other components in these hydrolysates.

Inhibitory compounds may be generated during the pretreatment and hydrolysis of lignocellulosic biomass. These include degradation products of sugars, such as the aldehydes furfural and 5-hydroxymethyl furfural (HMF). The effects of 0 to 4 g l^-1 ^of furfural and HMF on the fermentability of glucose were tested in small flasks. Both *C. saccharolyticus *and *T. neapolitana *were inhibited by these compounds. *C. saccharolyticus *appeared to be more sensitive (Table 4). Actively growing cells of *C. saccharolyticus *fully metabolized furfural to mainly furfuryl alcohol (>80%). However, at 4 g l^-1^, hardly any growth was observed and the furfural was no longer converted. Similar results were obtained with HMF, although the conversion products were not identified. *T. neapolitana *also metabolized furfural and HMF. Furfural was partly converted to furfuryl alcohol, while other conversion products were not identified. Previous reports also showed inhibition of growth of other bacteria by similar concentrations of furfural and HMF (IC_50 _at 1 to 4 g l^-1^; [23,24]), but a stimulatory effect (up to 3 g l^-1^) on the growth of *Clostridium beijerinckii *has also been reported [25]. The reduction of the furaldehydes to alcohols has been mentioned earlier as a detoxification step under both aerobic and anaerobic conditions [23]. In yeast, alcohol dehydrogenases and xylose reductase were responsible for the reduction of furfural and HMF [26,27]. Bacterial enzymes involved in furaldehyde reduction have not been identified yet, but the reduction appeared to be constitutive with reduced nicotinamide adenine dinucleotide (phosphate) (NAD(P)H) acting as the electron donor [28]. The concentration of furaldehydes in hydrolysate I and II was less than 0.1 g l^-1 ^and, therefore, the inhibition of *T. neapolitana *was not caused by these compounds.

### Hydrolysate preparation on bench scale and fermentability

**Figure 1:**
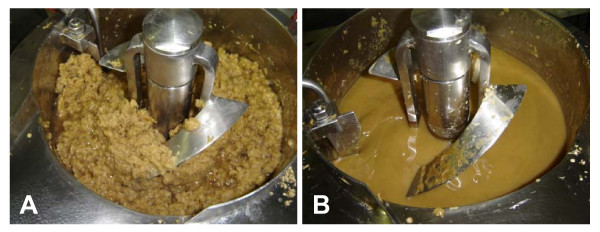
**Enzymatic hydrolysis of alkaline-pretreated *Miscanthus *on bench scale**. **(a) **The slurry immediately after addition of the enzyme preparation (GC 220 at a final loading rate of 15 IFPU per g dry matter). **(b) **After 24 h of hydrolysis. *Miscanthus *was pretreated with 9% NaOH (w w^-1^) for 6 h at 75°C.

The best fermentability was observed with hydrolysate IV. This batch was prepared from NaOH-pretreated *Miscanthus *that was washed with water to remove a large part of the alkali (and solubilised lignin), thus reducing the amount of acetic acid needed for pH adjustment. In order to validate this method and the process conditions at the bench scale, 1.35 kg of *Miscanthus *was cut and sieved to pieces of circa 1 cm, and subsequently treated with 9% NaOH (w w^-1^) for 6 h at 75°C in a 10 l stirred tank reactor. After dewatering, the pH of the slurry was adjusted with acetic acid, and the pulp was enzymatically hydrolyzed by using a fed-batch procedure. The viscosity of the slurry with 27.5% dry matter was rapidly reduced during the hydrolysis process (Figure 1). The clarified hydrolysate contained 38.3 g l^-1 ^of monosaccharides (Table 5). The conversion efficiency was comparable to the small-scale experiment (hydrolysate IV), despite the shorter period of alkali treatment (6 h instead of 16 h), and lower pretreatment temperature (75°C instead of 85°C). The total amount of monosaccharides in the hydrolysate increased by 19% after sulfuric acid treatment, indicating that other soluble sugars were present as di- and/or oligosaccharides (Table 5). More than half of the extra monomers were xylose. The incomplete degradation of the polysaccharides to monosaccharides is partly due to product inhibition of the hydrolytic enzyme activity. Xylan degradation to xylose appeared to be extra inefficient, most likely because the enzyme preparation was not optimally balanced for the complete degradation of xylans to xylose [29]. The contribution of carbohydrates to the total chemical oxygen demand (COD) of the hydrolysate was 73% (Table 5).

**Table 5:** Composition of *Miscanthus *hydrolysate prepared at the bench scale.

Components		g l^-1^	COD, g l^-1^
Monomeric sugars		38.3	40.0
	Glucose	26.8	
	Xylose	10.3	
	Arabinose	1.2	
Di- and/or oligosaccharides			7.6
Acetic acid		1.9	2.0
Lactic acid		1.8	1.9
Unknown components			13.8
*Total hydrolysate*			*65.3*

Pretreatment of 1.35 kg of milled *Miscanthus *(10 × 10 mm^2 ^sieve) was done at a solid content of 0.125 g g^-1 ^and 9% NaOH (w w^-1^) for 6 h at 75°C. Acetic acid was used for pH adjustment of the dewatered pulp prior to enzymatic hydrolysis (50°C for 24 h).

During enzymatic hydrolysis, the pH of the slurry decreased from the initial pH of 5.2 to 4.3. Besides the release of organic acids from the lignocellulosic material during hydrolysis, this decline can be ascribed to lactic acid formation by contaminating bacteria. The relative amount of other, unknown, organic compounds amounted to 21% of the total COD of the hydrolysate. Because of the mild process conditions (moderate temperature, ambient pressure, and no extreme acidic pH), sugar degradation products are not expected to be formed. Indeed, furfural and HMF were not detected in the hydrolysate (detection limit 10 mg l^-1^).

**Figure 2:**
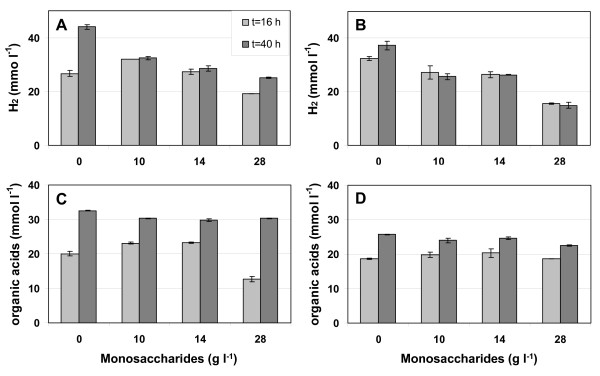
**Hydrogen and organic acid production by cultures of *C. saccharolyticus *and *T. neapolitana *grown on *Miscanthus *hydrolysate**. **(a) **and **(c) ***C. saccharolyticus*. **(b) **and **(d) ***T. neapolitana*. **(a) **and **(b) **Hydrogen production. **(c) **and **(d) **Organic acid (acetic and lactic acid) production. Measurements were done after 16 and 40 h after the start of the fermentation. Hydrolysate concentration is given in g monosaccharides l^-1^. In the control without hydrolysate, 10 g l^-1 ^of a glucose/xylose (7:3, w w^-1^) mixture was used as the carbon source. The error bars show the range about the mean of the values from two flasks.

The fermentability of this hydrolysate was tested in small flasks with *C. saccharolyticus *and *T. neapolitana*. The production of hydrogen and organic acids (acetate and lactate) was observed with hydrolysate concentrations up to 28 g l^-1 ^of monosaccharides (Figure 2). At 10 g l^-1^, hydrogen production from the hydrolysate by both thermophiles was less when compared to production from the corresponding pure sugars (glucose and xylose at 7 and 3 g l^-1^, respectively), and decreased further at higher hydrolysate concentrations. The production of organic acids by both bacteria was very similar for all hydrolysate concentrations, and comparable to that from pure sugars. Only the rate of organic acid production by *C. saccharolyticus *appeared to be lower at the highest hydrolysate concentration. The difference between hydrogen and organic acid production was due to increased lactate production at higher hydrolysate concentrations, which is not accompanied by hydrogen production. The production of lactate might be prevented in favour of acetate and hydrogen formation during fermentations under pH and hydrogen pressure (*p*H_2_) controlled conditions.

### Anaerobic batch fermentations under controlled conditions in a bioreactor

Fermentations under controlled conditions were done with *Miscanthus *hydrolysate and the glucose-xylose mixture using a continuously-stirred bioreactor (CSTR). The *p*H_2 _was kept below the critical concentration of circa 10 kPa [22] by flushing the culture medium with N_2_. Details of the fermentations are shown in Table 6. At concentrations of 10 and 14 g l^-1^, the consumption of pure sugars was completed within 22 to 34 h. At the end of the *T. neapolitana *fermentation some fructose was observed (<0.3 g l^-1^). Fructose might be the product of the activity of a xylose isomerase identified in this bacterium, which is able to catalyze the isomerisation of glucose to fructose [30]. Although *T. neapolitana *is able to grow on fructose, consumption is slow and usually incomplete (unpublished results). At a higher concentration of 28 g l^-1 ^of pure sugars, consumption by *C. saccharolyticus *was still incomplete after 71 h. The fermentation by *T. neapolitana *was stopped after 45 h because of brown-colouring of the culture medium due to the occurrence of Maillard reactions at the high fermentation temperature of 80°C. At that moment, hydrogen production was low (approximately 0.3% in the off gas).

**Table 6:** Fermentation parameters of 1 l batch cultures of *C. saccharolyticus *at 72°C and *T. neapolitana *at 80°C grown on a glucose/xylose (7:3, w w^-1^) mixture and on *Miscanthus *hydrolysate.

Mono-saccharides	Ferm. period	Consumption			Production						C-balance	COD-balance
			mmol l^-1^		mmol l^-1^					g l^-1^		
							
g l^-1^	*h*	Glucose	Xylose	COD	H_2_	CO_2_	Acetate	Lactate	COD	CDW		
*C. saccharolyticus*												
Glc/Xyl												
10	28	39.3	18.7		188	100	88.8	1.1		0.67	0.94	0.93
14	34	55.2	25.4		253	130	117	1.9	7.4	0.77	0.87	0.91
28	>71	77.4	54.8		293	171	136	3.7	53.1	0.84	0.66	0.79
Hydrolysate												
10	35	41.9	17.5	82.6	237	120	105	1.1		0.98	0.89	0.92
14	69	58.9	26.2	92.8	319	176	152	2.3		1.04	0.92	0.92
28	>71	52.4	41.5	97.9	244	153	125	4.5		1.01	0.74	0.88

*T. neapolitana*												
Glc/Xyl												
10	22	38.2	18.6		178	93.6	82.3	3.9		0.97	0.96	0.94
14	33	54.4	24.4		240	131	105	5.9	15.8	0.74	0.87	0.91
28	>45	66.7	21.3		189	113	89.9	14.2	58.4	0.42	0.81	0.90
Hydrolysate												
10	26	40.8	16.0	80.3	198	102	88.0	9.6		1.27	0.89	0.92
14	39	55.8	22.2	94.3	285	154	124	11.2		1.02	0.88	0.91
28	>45	42.9	22.8	-^a^	82.0	62.2	45.2	11.2	124	0.46	0.89	0.96

^a^Due to Maillard reactions and thus non-defined product formation, no net chemical oxygen demand (COD) consumption was measured.

**Figure 3:**
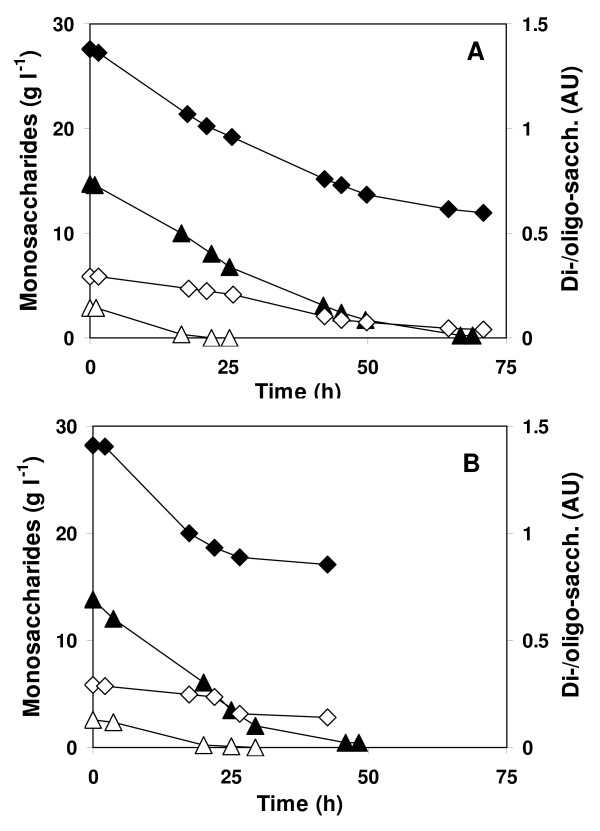
**Consumption of carbohydrates by cultures of *C. saccharolyticus *and *T. neapolitana *grown on *Miscanthus *hydrolysate**. Monosaccharide consumption (filled symbols) and consumption of di- and/or oligosaccharides (open symbols) by *C. saccharolyticus ***(a) **and *T. neapolitana ***(b) **in a medium containing *Miscanthus *hydrolysate. The hydrolysate concentration was at 14 (triangles) and 28 (diamonds) g monosaccharides l^-1^.

With low hydrolysate concentrations, the duration of the fermentation was prolonged by a few hours as compared to fermentations on pure sugars, except for the 14 g l^-1 ^fermentation by *C. saccharolyticus*, where the sugar consumption was significantly retarded. At the highest hydrolysate concentration, substrate consumption by *C. saccharolyticus *was incomplete and *T. neapolitana *cultures were severely coloured brown. Both thermophiles utilized other organic compounds in the hydrolysate besides glucose and xylose. Based on COD measurements, the extra consumption was circa 20%. Among the organic compounds were arabinose (approx. 3%), and di- and/or oligosaccharides. The latter were consumed within the first 25 h of the fermentations at a hydrolysate concentration of 14 g l^-1 ^of monosaccharides (Figure 3). The consumption of di- and/or oligosaccharides also occurred at the highest substrate concentration, but was initially less rapid.

**Figure 4:**
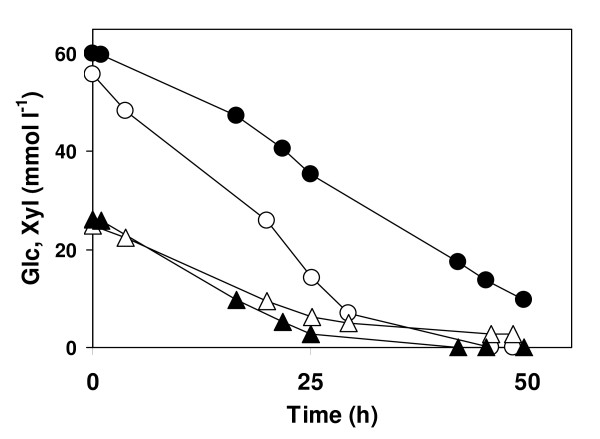
**Simultaneous consumption of monosaccharides by cultures of *C. saccharolyticus *and *T. neapolitana *grown on *Miscanthus *hydrolysate**. Consumption of glucose (circle) and xylose (triangle) by *C. saccharolyticus *(filled symbols) and *T. neapolitana *(open symbols) in a medium containing *Miscanthus *hydrolysate. The monosaccharide concentration was 14 g l^-1^.

Both thermophiles simultaneously consumed glucose and xylose, but *T. neapolitana *preferred glucose, while *C. saccharolyticus *consumed both sugars at a very similar rate (Figure 4). It has been shown previously that *C. saccharolyticus *has a preference for xylose if both sugars are present at equal concentration [31]. At higher glucose/xylose ratios, such as in the *Miscanthus *hydrolysate, a preference for xylose was no longer observed.

**Figure 5:**
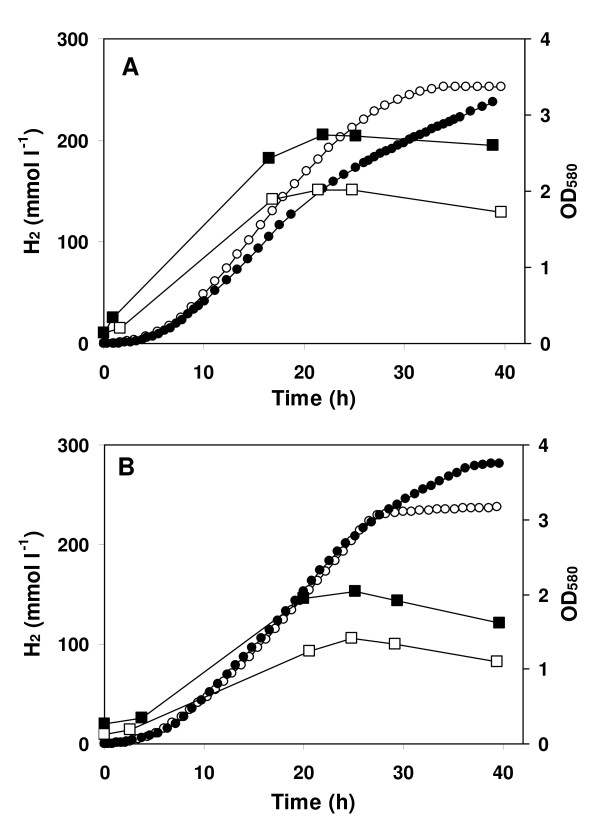
**Hydrogen production by cultures of *C. saccharolyticus *and *T. neapolitana *grown on pure sugars and *Miscanthus *hydrolysate**. Growth (squares) and hydrogen production (circles) in cultures of *C. saccharolyticus ***(a) **and *T. neapolitana ***(b) **grown on a glucose/xylose (7:3, w w^-1^) mixture (open symbols) or *Miscanthus *hydrolysate (filled symbols). The monosaccharide concentration was 14 g l^-1^.

The main products of the fermentations were hydrogen, acetate and CO_2 _(Table 6). The highest amount of hydrogen, 319 mmol l^-1 ^or 7.1 l l^-1^, was produced by *C. saccharolyticus *in a fermentation of 14 g l^-1 ^monosaccharides from the *Miscanthus *hydrolysate. In general, hydrogen and acetate production by *C. saccharolyticus *were higher than by *T. neapolitana*. For the fermentations at lower sugar concentrations, this was partly due to the slightly higher lactate production by *T. neapolitana *as compared to *C. saccharolyticus*. Lactate production by *C. saccharolyticus *was very low in fermentations on pure sugars, as well as on the hydrolysate. *T. neapolitana *produced a little more lactate on the hydrolysate than on pure sugars. Both bacteria grew to higher densities on the hydrolysate than on pure sugars (Table 6 and Figure 5), which might be due to the supplementation of the medium with some nutrients originating from the hydrolysate.

On pure sugars at 10 g l^-1^, carbon balances were more than 90%. At increased substrate concentrations the carbon balances often became less, indicating that more non-identified products were formed. Part of these products were volatile components, since COD balances, including the gaseous hydrogen, were still incomplete and volatile components in the gas stream were not collected. The large amount of non-defined products in the *T. neapolitana *fermentation at high hydrolysate concentration was due to the Maillard reactions.

### Hydrogen yield and productivity

The molar yields of products on the consumed substrates are shown in Table 7. The hydrogen yields (*Y*_H2_) at low-sugar concentrations were generally more than 3 mol H_2 _per mol hexose, with a maximum of 3.4 mol per mol hexose obtained in fermentations with *C. saccharolyticus*. For *C. saccharolyticus*, the hydrogen yield after growth on hydrolysate was equally high as that from pure sugars. The yields with cultures of *T. neapolitana *on hydrolysate varied between 89% to almost 100% of the yield on pure sugars. The yield dropped substantially at the highest substrate concentrations. The biomass yield (*Y*_xs_) of both thermophiles also decreased with increasing substrate concentrations.

The increased production of compounds other than biomass and acetate apparently was not accompanied by hydrogen production. In all fermentations, except those with Maillard reactions, the hydrogen to acetate molar ratio varied between 2.0 to 2.2, which is close to the theoretical value of 2. The CO_2 _to acetate molar ratio was circa 1.1 at the low substrate concentrations. This ratio increased to 1.3 at the highest substrate concentration, which suggests that a decarboxylation step is involved in the metabolic pathways for the other products. One of these products could be ethanol. Both thermophiles are able to form ethanol, but it is often lost in the gas stream at the high fermentation temperatures because of its volatility.

**Table 7:** Molar yields and maximal volumetric hydrogen productivity (*Q*_H2, max_) of *C. saccharolyticus *and *T. neapolitana *batch cultures grown on a glucose/xylose (7:3, w w^-1^) mixture and on *Miscanthus *hydrolysate.

Mono-saccharides	Molar yield				Q_H2, max_
	mol (mol C6)^-1^			g (mol C6)^-1^	
			
g l^-1^	*Y*_H2_	*Y*_Ac_	*Y*_CO2_	*Y*_xs_	mmol l^-1 ^h^-1^
*C. saccharolyticus*					
Glc/Xyl					
10	3.4	1.6	1.8	12.2	12.0
14	3.3	1.5	1.7	10.1	13.0
28	2.4	1.1	1.4	6.8	9.7
Hydrolysate					
10	3.4	1.5	1.7	14.0	12.6
14	3.3	1.6	1.8	10.8	10.4
28	2.4	1.2	1.5	9.8	6.2

*T. neapolitana*					
Glc/Xyl					
10	3.3	1.5	1.7	18.1	14.5
14	3.2	1.4	1.7	9.9	11.9
28	2.5	1.2	1.5	5.0	6.1
Hydrolysate					
10	2.9	1.3	1.5	18.8	13.1
14	3.2	1.4	1.7	11.3	12.3
28	2.0	1.1	1.5	7.4	5.4

Most of the hydrogen was produced during the exponential growth phase, but even when the cell density decreased, hydrogen was still produced, although often at a lower rate (Figure 5). The hydrogen production rate by *T. neapolitana *was very similar on pure sugars and on *Miscanthus *hydrolysate. The productivity by *C. saccharolyticus *on hydrolysate was lower than the productivity on pure sugars (Figure 5), which is in line with the lower sugar consumption rate. Apparently, *C. saccharolyticus *is inhibited by the hydrolysate.

**Figure 6:**
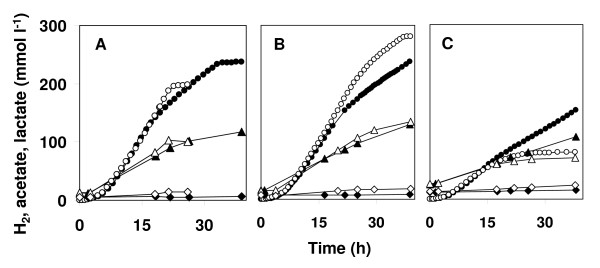
**Effect of *Miscanthus *hydrolysate concentration on the production of hydrogen, acetate and lactate by cultures of *C. saccharolyticus *and *T. neapolitana***. Production of hydrogen (circle), acetate (triangle) and lactate (diamond) in cultures of *C. saccharolyticus *(filled symbols) and *T. neapolitana *(open symbols) grown on *Miscanthus *hydrolysate at concentrations of 10 **(a)**, 14 **(b) **and 28 **(c) **g monosaccharide l^-1^.

Initially, hydrogen productivity by both thermophiles on low concentrations of *Miscanthus *hydrolysate was comparable, but after circa 20 h the productivity by *C. saccharolyticus *declined as compared to that by *T. neapolitana *(Figure 6). At this point, the cell density of *C. saccharolyticus *started to decrease (Figure 5). The cell density of *T. neapolitana *decreased after circa 22 h, but *T. neapolitana *appeared to be able to maintain reasonable hydrogen productivity as long as the substrate was not completely consumed (Figure 6(b)). The reason for the early decrease in cell density of the thermophiles while substrate is still available is unknown. Some nutrients may have become limiting or the bacteria sense a high cell density and react by arresting growth. When the growth of *C. saccharolyticus *stopped, the concentration of organic acids (acetate and lactate) was approximately 90 mM. This is far below the concentration of acetate (150 to 175 mM), where cell lysis starts to dominate [22]. At the highest hydrolysate concentration of 28 g l^-1 ^monosaccharides, hydrogen productivity by both thermophiles was low. After circa 20 h, hydrogen and organic acid production by *T. neapolitana *virtually stopped.

The maximum volumetric hydrogen productivity (*Q*_H2, max_) occurred at the late exponential growth phase of the thermophiles. The highest *Q*_H2, max _of 14.5 mmol l^-1 ^h^-1 ^was observed with *T. neapolitana *that was fermenting 10 g l^-1 ^of pure sugars (Table 7). The maximum *p*H_2 _measured in the off gas was 4.6 kPa. In the *C. saccharolyticus *fermentations, hydrogen pressures of circa 4 kPa were found, which is significantly lower than the critical value of 10 to 20 kPa. For *T. neapolitana *the critical value has not been established yet.

## Conclusion

Cultures of *C. saccharolyticus *and *T. neapolitana *produced hydrogen and acetic acid as the main organic acid during the fermentation of sugars in hydrolysates prepared from the lignocellulosic energy crop *Miscanthus*. The mild conditions of alkaline treatment enabled the optimized pretreatment protocol to be compatible with the thermophilic fermentations. The formation of potential inhibitors, for example, sugar degradation products, was negligible. For future applicability on an industrial scale this one-stage process needs to be further optimized with respect to alkali usage, for instance by regeneration and recycling of the alkali agent.

Both thermophiles, *C. saccharolyticus *and *T. neapolitana*, appeared to be able to simultaneously and completely utilize all soluble monomeric C5 and C6 sugars, di- and oligosaccharides up to a total sugar concentration of 17 g l^-1^. The capacity of co-fermenting glucose and xylose by *C. saccharolyticus *was recently confirmed by whole-genome transcriptome analysis [32]. Simultaneous and complete substrate utilization from complex feedstocks, such as the hydrolysates of lignocellulosic biomass, will add to an energy-efficient process and is a major advantage in industrial scale production facilities.

The observed hydrogen yields resulting from the thermophilic fermentations were 74 to 85% of the theoretical value of 4 mol per mol hexose in fermentations with circa 17 g l^-1 ^total sugars. These are amongst the highest hydrogen yields obtained in the fermentation of sugars in lignocellulosic hydrolysates reported to date. *C. saccharolyticus *offers the advantage of a nearly 10% higher hydrogen yield during growth on *Miscanthus *hydrolysates as compared to *T. neapolitana*. However, the rate of substrate consumption and hydrogen production by *T. neapolitana *was higher. *C. saccharolyticus *seemed to be hampered by the increase in ionic strength of the culture medium during fermentation and showed lower hydrogen productivity in the stationary phase. Because *T. neapolitana *is a moderately halophilic organism, it tolerates conditions with higher ionic strength, and possibly with higher osmolalities.

Cultures growing on *Miscanthus *hydrolysates had low volumetric hydrogen productivity with a maximum of 13 mmol l^-1 ^h^-1 ^and a mean productivity of circa 7 mmol l^-1 ^h^-1^. Higher volumetric hydrogen productivities have been reported for systems with higher cell densities, such as the carrier-induced granular sludge bed (CIGSB) bioreactor. Productivities of more than 300 mmol H_2 _l^-1 ^h^-1 ^have been observed, but the hydrogen yield of circa 50% on pure sucrose was low [33].

The COD reduction of the culture medium containing *Miscanthus *hydrolysate was limited to 30% because the carbohydrates were only partially oxidized to organic acids. More hydrogen can potentially be produced from the organic acids in the effluent by photobacteria, which use light as an extra energy source. Theoretically, another 4 moles of H_2 _and 2 moles of CO_2 _can be produced from acetic acid. The first experiment was done with supernatant that was obtained from a culture of *T. neapolitana *after growth on *Miscanthus *hydrolysate. *Rhodobacter capsulatus*, a purple non-sulfur bacterium, was able to grow and produce hydrogen on the supernatant, which was diluted twice, and supplemented with Fe(III)-citrate [34]. The yield of the two fermentations was circa 4.5 moles of hydrogen per mole hexose, that is, 37% of the theoretical value of 12 moles of hydrogen per mole of glucose. Further research is aimed at improving the hydrogen production efficiency of this combined thermophilic and photoheterotrophic fermentation [35].

## Methods

### Biomass pretreatment and enzymatic hydrolysis

*Miscanthus giganteus *was collected in the spring of 2004 from a location in Groningen, The Netherlands and consisted primarily of stems. The dry matter content of the harvested stems was circa 80 to 85% on a wet weight basis. The total carbohydrate and lignin content on a dry weight basis was 63% (including 42% glucose, 19% xylose, and 1% arabinose) and 23%, respectively.

Alkaline pretreatment experiments at the lab scale (2 l pulp mixer, Quantum Mark V reactor) were done with milled *Miscanthus *(Retsch SM 200 mill equipped with a 2 mm screen). An amount of 225 g of biomass (circa 200 g dry matter) was pretreated with 9 to 12% Ca(OH)_2 _or NaOH (w w^-1 ^dry matter, for details see Tables 1 and 2) at a solid:liquid ratio of 0.125 (w w^-1^). Pretreatment was done at 85°C for 16 h. One batch of pretreated *Miscanthus *was washed three times with demineralised water. Prior to enzymatic hydrolysis, the pH of the pretreated material was adjusted to 4.8 to 5.1 with 17% phosphoric, 20% sulfuric or 25% acetic acid (v v^-1^). Enzymatic hydrolysis of the pretreated biomass was done using commercial enzyme preparations (Cellubrix and Novozymes 188 from Novozymes, Bagsvaerd, Denmark and GC 220 from Genencor, Rochester, NY, USA). The amount of enzyme added per 100 g dry matter was 28, 9, and 13 ml of Cellubrix, Novozymes 188 and GC 220, respectively (for details see Table 2). The enzyme concentration was selected to warrant a similar cellulase activity of 15 IFPU per g dry matter, on the basis of cellulase activity measurements [29]. Incubation was done at 50°C for 24 h.

A pretreatment and hydrolysis experiment was also done at the bench scale in a 10 l stirred vessel. An amount of 1.35 kg (circa 1 kg dry matter) of milled *Miscanthus *(Pallmann type PS 3–5 knife mill equipped with a screen of 10 mm × 10 mm square opening) was added to the vessel, together with NaOH, under continuous mixing (90 rpm). The amount of NaOH was 9% (w w^-1^) at a solid:liquid ratio of 0.125 (w w^-1^). The pretreatment was done for 6 h at 75°C. The material was then dewatered using a manual piston press to make a slurry of 275 g dry matter l^-1^. The pH of the remaining viscous pulp was adjusted to 5 using a 20% (v v^-1^) acetic acid solution. Enzymatic hydrolysis was done by a fed-batch procedure of GC 220 addition. The incubation was at 50°C for 24 h.

Hydrolysates were collected after neutralization of the pH and removal of the solids of the enzymatically hydrolyzed material by centrifugation. The hydrolysates were stored at -15°C until use.

### Microorganisms and medium

*C. saccharolyticus *DSM 8903 and *T. neapolitana *DSM 4359 were obtained from the Deutsche Sammlung von Mikroorganismen und Zellkulturen (DSMZ). The culture medium consisted of (per l) KH_2_PO_4 _0.3 g, K_2_HPO_4 _0.3 g, MgCl_2_.6H_2_O 0.4 g, NH_4_Cl 0.9 g, yeast extract 1.0 g, cysteine-HCl 0.75 g, FeCl_3_.6H_2_O 2.5 mg, SL-10 trace elements 1 ml, and resazurin 0.5 mg. The pH was adjusted to 7.0 at room temperature. NaCl (5 g per l) was added to the culture medium of *T. neapolitana*. The culture medium for flask experiments was supplemented with 50 mM 4-morpholine propanesulfonic acid (MOPS) to increase the buffering capacity of the medium. A mixture of glucose and xylose (7:3, w w^-1^) or *Miscanthus *hydrolysate sugars were used as the carbon source. The medium was made anoxic by flushing with N_2_. The experiments were carried out under non-sterile conditions. *C. saccharolyticus *and *T. neapolitana *were grown at 72 and 80°C, respectively.

The fermentability of hydrolysates was tested using flasks of 118 ml with 20 ml culture medium under a nitrogen atmosphere. The total monosaccharide concentration was 10 g l^-1 ^coming from the pure sugar mixture, the hydrolysate, or a combination of both. The flasks were inoculated with 5% (v v^-1^) of a preculture that was grown overnight on the same pure sugar mixture. After 16 and 40 h, samples were withdrawn from the headspace (duplicate gas sample of 0.2 ml) and the culture medium (single sample of 1 ml) for analyses of the hydrogen production, cell density, pH, and organic acid production. The experiments were carried out in duplicate (two flasks per condition). The inhibition of compounds was tested using the same method, except that 10 g l^-1 ^of glucose was used as the carbon source.

Batch fermentations under controlled conditions were carried out in a jacketed 2 l bioreactor (Applikon, Delft, The Netherlands) with a working volume of 1 l. The pH was controlled at circa 6.8 (measured at room temperature) by automatic addition of 2 N NaOH. The cultures were continuously stirred at 350 rpm and sparged with N_2 _at 7 l h^-1^. Inoculation was done by adding 10% (v v^-1^) of a preculture that was grown overnight on the glucose/xylose mixture. A fermentation was considered to have ended when the hydrogen concentration was less than 0.2% in the off gas. Samples of 7 ml were regularly taken from the culture medium for measurement of the cell density and substrate and product analyses. Hydrogen and CO_2 _were measured in the off gas each hour. Data are from one representative fermentation per condition.

### Analytical methods

Determination of the acid-soluble and acid-insoluble lignins was performed according to the Tappi method [36]. Organic acids were analyzed by high performance liquid chromatography (HPLC) using a Shodex ionpak KC811 column (Waters, The Netherlands), as described earlier [37]. Monosaccharides, di- and oligosaccharides, furfural, HMF and furfuryl alcohol were analyzed by HPLC using an Altech IOA-1000 column at 90°C, with 3 mM sulfuric acid as the mobile phase (0.4 ml per min), followed by detection by differential refractometry. Fructose was determined enzymatically (Megazyme International Ireland Ltd, Bray, Ireland). Hydrogen in the headspace of the serum bottles and hydrogen and CO_2 _production in the bioreactors were measured as previously described [37]. COD measurements of the culture medium and hydrolysates were done using the LCK test kit 014 of Hach Lange (Düsseldorf, Germany). The hydrolysis of di- and oligosaccharides in hydrolysates to monomeric sugars was done by the addition of concentrated sulfuric acid (95 to 97%) to a final concentration of 1 M and incubation at 95°C for 1 h. The optical density of the cultures was measured against a water blank at 580 nm after dilution of the culture broth with deionised water. The cell dry weight was determined from the highest value of the optical densities using the relation CDW (g l^-1^) = (0.377 × *OD*_580_) + 0.011 for *C. saccharolyticus *[37] and CDW (g l^-1^) = (0.528 × *OD*_580_) for *T. neapolitana*. The molecular weight of *T. neapolitana *was assumed to be the same as the measured value for *C. saccharolyticus*, that is, 24.6 g (mol C)^-1 ^[37].

### Yield and productivity

The amount of consumed substrate, including non-defined organic compounds, was used for calculating product yields. Yields were expressed as mol product per mol C6 sugar. Because the theoretical hydrogen and acetate yields per C-mol are equal for glucose and xylose [8], the molar amount of xylose was converted to a molar amount of hexose. The consumed unknown organic compounds were determined from COD measurements. They were considered to be carbohydrates with the same product yield as for glucose and xylose. COD in mmol O_2 _l^-1 ^was converted to mmol hexose l^-1^, according to the equation C_6_H_12_O_6 _+ 6O_2 _→ 6CO_2 _+ 6H_2_O. The maximum volumetric hydrogen productivity was calculated from the time interval with the highest percentage of hydrogen in the off gas.

## Competing interests

The authors declare that they have no competing interests.

## Authors' contributions

TdV carried out most of the chemical inhibitor studies and analyzed the chemical composition of the hydrolysate, and drafted the manuscript. RRB performed the alkaline pretreatment, enzymatic hydrolysis experiments and the analysis of hydrolysates, and helped to draft the manuscript. MAWB carried out the 1 litre-scale fermentations. MHL carried out the hydrolysate fermentability tests and some of the chemical inhibitor studies. AEM helped to draft the manuscript. PAMC participated in the coordination of the study and helped to draft the manuscript. All the authors read and accepted the final manuscript.
